# Exploring Metal Ions as Potential Antimicrobial Agents to Combat Future Drug Resistance in *Mycoplasma bovis*

**DOI:** 10.3390/microorganisms13010169

**Published:** 2025-01-15

**Authors:** Mauida F. Hasoon Alkhallawi, Majed H. Mohammed, Farhid Hemmatzadeh, Kiro Petrovski

**Affiliations:** Australian Center for Antimicrobial Resistance Ecology, School of Animal & Veterinary Sciences, The University of Adelaide, Rose Worthy Campus, Mudla Wirra Rd., Roseworthy, SA 5371, Australia; majed.al-saegh@adelaide.edu.au (M.H.M.); farhid.hemmatzadeh@adelaide.edu.au (F.H.)

**Keywords:** conventional antimicrobial, colloidal silver, metal ions, combat resistance

## Abstract

The rise in antimicrobial resistance (AMR) in *Mycoplasma bovis* underscores the urgent need for alternative treatments. This study evaluated the minimal inhibitory concentrations (MICs) of four metal ions (cobalt, copper, silver, and zinc) and colloidal silver against 15 clinical *M. bovis* isolates, alongside conventional antimicrobials (florfenicol, tetracycline, tulathromycin, and tylosin). Colloidal silver demonstrated the most effective antimicrobial activity, inhibiting 81.25% of isolates at 1.5 mg/L, while silver inhibited 93.7% of isolates at concentrations above 1.5 mg/L. Copper exhibited notable efficacy, inhibiting 37.5% of isolates at 1.5 mg/L, with a small proportion responding at 0.1 mg/L. Cobalt and zinc displayed variable activity, with MIC values ranging from 0.7 to 12.5 mg/L. In contrast, conventional antimicrobials showed limited effectiveness: tetracycline inhibited 31.25% of isolates at ≥16 mg/L, tylosin inhibited 25% at 16 mg/L, and tulathromycin MICs ranged from 0.5 to 8 mg/L. Time–kill assays revealed a reduction in *M. bovis* viability after eight hours of exposure to silver and colloidal silver, though higher concentrations (4×–8× MIC) were required for complete eradication. These findings highlight the significant potential of colloidal silver and copper as alternatives for treating *M. bovis* infections and combating AMR. Further research is essential to explore their standalone and synergistic applications for therapeutic use.

## 1. Introduction

*Mycoplasma bovis* is a significant veterinary medicine pathogen, posing considerable challenges to livestock health worldwide. Belonging to the class Mollicutes, these bacteria lack a cell wall, rendering them resistant to conventional antimicrobials targeting cell wall synthesis (e.g., beta-lactams). As a result, *M. bovis* infections often present a treatment challenge, leading to substantial economic losses in affected livestock populations [1]. In this introduction, we briefly delve into the background of *M. bovis*, its pathogenicity, diagnostic challenges, and the implications it holds for veterinary medicine [2]. Not only is the interaction of *M. bovis* with host cells a factor of its pathogenicity, but also it influences its response to antimicrobials. In the absence of an effective vaccine for the major bovine respiratory disease (BRD) bacterial pathogens, including *M. bovis,* antimicrobial therapy remains the main treatment [3]. Conventional antimicrobials such as phenicols, tetracyclines, and macrolides have been the cornerstone in treating *M. bovis* infections. These antimicrobials typically target protein synthesis and other bacterial biosynthetic pathways [4]. Antimicrobial resistance (AMR) occurs when pathogens no longer respond to medicines, making infections difficult to treat and increasing the risk of disease spread, severe illness, and death [5]. Traditional antimicrobials have been the mainstay of managing bacterial infections in both human and veterinary medicine. However, the overuse and misuse of these drugs in humans, animals, and agriculture are assumed to have accelerated the development of resistant strains. For *M. bovis*, this means that some of the standard treatments are becoming less effective, necessitating higher doses or the use of alternative, often more expensive or less-understood antimicrobials [1]. This not only increases the cost of treatment but also poses a risk of undesirable side effects in animals and potentially humans through the consumption of their products.

The resistance mechanisms are often linked to genetic mutations that alter drug targets or enhance efflux pumps, further diminishing the effectiveness of conventional treatments [6]. The development of resistance in BRD pathogens, such as *M. bovis,* is a major reason for poor response to treatment and is a growing issue worldwide [1,6]. With the development and spread of AMR, there are higher chances of treatment failure, leading to prolonged outbreaks and greater economic losses. The ability of *M. bovis* to develop resistance complicates the management of herd health and necessitates more stringent biosecurity and management measures [7]. Therefore, the emerging AMR in *M. bovis* isolates requires the exploration of alternative antimicrobial actives, including but not limited to metals. Some metals have been reported as promising agents in combating bacterial infections, especially silver (Ag), copper (Cu), iron (Fe), and zinc (Zn). They exhibit potent antibacterial properties by disrupting various cellular processes essential for bacterial survival and replication. The mechanisms underlying the antibacterial action of these metal ions are diverse and multifaceted, often involving the generation of reactive oxygen species (ROS), disruption of cell membranes, interference with protein and DNA functions, and modulation of metal ion homeostasis [8,9].

The mechanisms of copper antimicrobial activity are related to ion (Cu^++^) toxicity, leading to compromised structural integrity of cellular components, including disruption of cellular membrane integrity, disruption of microbial metabolic pathways, and activation of microbial stress responses. Compromised structural integrity of cellular components, related to copper exposure, may occur due to the generation of ROS. This leads to oxidative damage of cellular lipids, nucleic acids, and proteins, ultimately resulting in cell death [10,11,12]. The disruption of microbial cell membranes leads to increased permeability and leakage of cellular contents [13]. Disruption of microbial metabolic pathways may occur through copper binding to active sites or cofactors in essential enzymatic processes. Finally, microbial stress responses resulting from exposure to copper lead to the upregulation of defense mechanisms that can damage bacterial cells with prolonged stress, ultimately causing cell death [14].

Silver is the metal with the greatest association with antimicrobial activity, both historically and currently [15]. Various medical products include silver, such as bandages, ointments, and catheters [16]. Silver nanoparticles and colloidal silver (an alloy with the antimicrobial sulfadiazine) are commonly used [17]. The action of silver ions (Ag^+^) at the microbial cellular level and their potential as an alternative to conventional antimicrobials are critical areas of interest, especially in the face of rising AMR [16]. Despite the different forms, ions are generally believed to be the active component, and the antibacterial activity varies with size, shape, and surface characteristics (including surface coatings) of the silver. However, these differences are likely due to changes in the release kinetics of Ag^+^ and not because of the particles themselves [18]. The mechanisms of Ag⁺ toxicity involve compromising the structural integrity of cellular components, disrupting the cell membrane, and interfering with microbial metabolic pathways [19,20]. Compromised structural integrity of cellular components, related to silver exposure, may occur due to the high affinity to sulphur-containing compounds in the bacterial cells, particularly thiol groups in sulphur-containing amino acids. This results in damage to nucleic acids and proteins, ultimately resulting in cell death [21] or in the prevention of bacterial replication. The disruption of the microbial cell membranes leads to increased permeability and leakage of cellular content and/or penetration of environmental materials, ultimately leading to bacterial death [21,22].

Cobalt and zinc ions have also re-emerged as promising candidate agents for use as antimicrobials. The antimicrobial properties are related to the toxicity of ions (Co^++^ or Zn^++^), leading to disruption of cellular membrane integrity and disruption of bacterial metabolic pathways, and triggering a microbial stress response [21]. Additionally, cobalt and/or zinc ions, when combined with conventional antimicrobials, may boost effectiveness through increased uptake, potentiation of activity, or another synergism [8,23]. Cobalt is also a crucial component of vitamin B_12_, playing a vital role in bacterial metabolism. Synthetic analogs of vitamin B_12_, containing cobalt, may disrupt bacterial metabolic pathways [24].

The exploration of these metal ions as potential alternatives to antimicrobials is not without its challenges. Concerns over toxicity, environmental impact, and the development of resistance necessitate careful considerations [25]. Copper-based compounds, for example, have demonstrated low toxicity in in vivo models like *Galleria mellonella* larvae when injected directly into the haemocoel [25]. Similarly, silver compounds, while exhibiting low toxicity in controlled doses, present risks of acute and chronic adverse effects, such as gastrointestinal irritation and argyria, when overexposed. Therefore, due to toxicity concerns, their primary use is in topical formulations rather than systemic applications, such as silver sulfadiazine [25,26]. Additionally, the environmental impact of heavy metals should not be ignored.

Recent studies [27,28] have suggested that efflux pump mechanisms play a crucial role in bacterial responses to metal ions by regulating intracellular concentrations and mitigating their antimicrobial effects. Overexpression or mutations in efflux pump genes can enhance the expulsion of metal ions, potentially reducing their efficacy as antimicrobial agents. However, these resistance mechanisms vary significantly among bacterial species and isolates [29]. However, the potential benefits, including the reduced likelihood of resistance development and their broad-spectrum activity, make the pursuit of understanding and harnessing these metal ions as antimicrobials a compelling area of study [8].

The present study aimed to explore the efficacy of metal ions in inhibiting the in vitro growth of clinical isolates of *M. bovis* that have failed the conventional treatment, in comparison to conventional antimicrobials, by determining the minimum inhibitory concentrations (MICs) of various metal ions and time–kill kinetics assays. This comparative analysis not only highlights the antimicrobial potential of metal ions but also provides a foundation for future investigations into their mechanisms of action and application in livestock management practices.

## 2. Materials and Methods

### 2.1. Mycoplasma Isolates (Identification and Culture)

A total of 15 *M. bovis* isolates were obtained from government and private veterinary service laboratories in Australia and archived at the School of Animal and Veterinary Science, The University of Adelaide. The isolates were collected from cattle showing signs of the BRD complex and were either treated (*n* = 5), euthanized (*n* = 4), or had prolonged exposure to tetracycline-based metaphylactic treatment (*n* = 6) from 2021 to 2023. The identity of the isolates was confirmed by polymerase chain reaction (PCR) using specific primers for the Deoxy-ribodipyrimidine photolyase (uvrC) gene at F: 5′ AAG TTG AAG TTG ACC GGT TTG 3′ and R: 5′ TCC ATA TTT GGA CCT AGT CCT TT 3′ [30]. To confirm the identification, the PCR products were sent for sequencing (AGRF—https://www.agrf.org.au accessed on 9 December 2024).

*Mycoplasma bovis* Type Strain PG45 (ATCC 25523) was used as a reference strain for quality control assessment. The isolates (including the reference type strain) were cultured using Eaton broth/agar, prepared in-house with PPLO broth (Thermo Fisher Scientific, Melbourne, Australia) supplemented with 1% (*w*/*v*) yeast extract, glucose, sodium pyruvate, and 20% (*v*/*v*) horse serum (Sigma-Aldrich, Melbourne, Australia). Broths were incubated at 37 °C in a 5% CO_2_ atmosphere for 3–5 days and then plated to inspect for the typical fried-egg colony morphology of *Mycoplasma*. Before testing, each cultured broth was diluted to a known concentration (10^5^ to 10^6^ CFU/mL) following standard laboratory procedures for veterinary mollicutes [31].

### 2.2. Antimicrobial Susceptibility Testing

The methodology for determining the minimum inhibitory concentrations (MICs) of conventional antimicrobials and metal ions involved a modified broth microdilution method adapted from the standards recommended by the Clinical and Laboratory Standards Institute (CLSI, [32]) and adjustments proposed by Hasoon et al. [6]. Initially, *Mycoplasma* suspensions were prepared in Eaton broth, with the optical density adjusted to 0.08–0.12 at OD 600 nm using Spectrophotometer [Eppendorf BioPhotometer^®^, Hamburg, Germany]. These suspensions were then further diluted to achieve a final concentration of approximately 10^6^ CFU/mL in each well of sterile 96-well polystyrene microtiter plates (Nunclon™; Thermo Fisher Scientific, Australia), with 50 µL of Eaton medium dispensed into each well. Metal powders were dissolved in 2% nitric acid and further diluted in Eaton broth to achieve the desired concentrations for stock solution preparation. The stock solutions of conventional antimicrobials (florfenicol, tetracycline, tulathromycin, and tylosin) and metals or the alloy (cobalt, copper, silver, zinc, and colloidal silver; metal hereafter) were prepared at a 10X concentration and stored at −80 °C until use. The antimicrobials were tested at concentrations ranging from 0.25 to 128 mg/L using two-fold serial dilutions, while metal ions were tested at concentration ranges between 0.1 mg/L to 100 mg/L. Each microtiter plate was set up by adding serial dilutions of each tested agent (antimicrobial or metal), followed by 50 µL of the *Mycoplasma* inoculum (10^6^ CFU/mL) to each well. To validate the assay’s accuracy, the plates included both a positive control medium (without antimicrobials or metals) and a negative control medium (without *Mycoplasma* suspension). The assay plates were sealed and incubated until complete growth was seen in positive control wells (~3 days) at 37 °C in a 5% CO_2_ environment. To ensure the reliability and reproducibility of results, all tests were conducted in triplicates.

#### Interpretation of the MIC Testing

MIC values were determined as the lowest concentration of the antimicrobial agent that completely inhibited visible growth. The MIC_50_ and MIC_90_ values (concentrations inhibiting 50% and 90% of isolates, respectively) were calculated. The reference type strain (PG45) was used to standardize the work by comparing our MICs with values reported earlier [6].

Due to the lack of breakpoints for veterinary *Mycoplasma* spp., interpretation as susceptible, intermediate, or resistant was not possible. Therefore, the MIC results for the three antimicrobial groups (macrolides, tetracyclines, and phenicol), and five metal agents (cobalt, copper, silver, colloidal silver, and zinc) tested in this study were interpreted based on MICs for the *M. bovis* reference type strain PG45 (ATCC^®^ 25523).

### 2.3. Metal Solvent Toxicity Testing

To ensure the 2% nitric acid used in metal stock solution preparation did not affect *Mycoplasma* growth, a separate toxicity test was conducted. Broths with and without nitric acid were incubated and examined for growth over 5–7 days at 37 °C in 5% CO_2_.

### 2.4. Time–Kill Kinetics Assay of Metal Ions

Time–kill kinetics assays for Co, Cu, Ag, Zn, and colloidal silver were carried out as previously described [33]. Briefly, selected *Mycoplasma* strains (those that exhibited high MIC to florfenicol, tetracycline, tulathromycin, and tylosin), together with PG45 ATCC strain, were prepared at an inoculum size of 1 × 10^6^ CFU/mL in a sterile Eaton broth medium, and seeded in 96-multiwell plates, followed by the addition of successive concentrations of each metal ion MIC (0.5× MIC, 1× MIC, 2× MIC, and 4× MIC), and then incubated (37 °C, 5% CO_2_). A total of 0.1 mL of cultures was taken at time intervals of 0, 8, 24, 48, and 72 h and spread onto Eaton agar plates and incubated at 37 °C for 48–72 h. The viable *M. bovis* cells were counted as CFU/mL, and data were reported for curve plotting. Each time–kill curve showed the percentage of *Mycoplasma* surviving over the time of development.

### 2.5. Statistical Analysis

To compare the inhibition percentages of different agents (antimicrobials and metals) against *M. bovis*, and to assess the time to kill *Mycoplasma* exposed to different concentrations (from 0.5 MIC to 8 MIC) of metals, ANOVA followed by Tukey’s HSD test was carried out in SAS version 9.4.

## 3. Results and Discussion

The current study aimed to assess the susceptibility of 15 Australian *M. bovis* isolates obtained from feedlot BRD cases to four antimicrobials (belonging to three different groups: macrolides, phenicol, and tetracyclines) and enable comparison with five metals (Ag, Cu, Co, Zn, and colloidal silver).

There is no certain specification of treatments considered yet for bovine mycoplasmas. However, The Australian Strategic and Technical Advisory Group on Antimicrobial Resistance (ASTAG) has developed a specific rating system for antimicrobials used in both human and veterinary settings according to their clinical importance, with the majority of antimicrobials registered for the treatment of BRD (amphenicols, macrolides, and tetracyclines) given a low importance rating [6]. Following the development of an antimicrobial stewardship program for Australian feedlots, a recent survey of antimicrobial use has shown an extensive reliance on these low-importance antimicrobials for treating BRD [3].

### 3.1. Mycoplasma Isolates Selection

Fifteen bovine Mycoplasma isolates were identified as *M. bovis* through PCR targeting a 106 bp fragment of the deoxy-ribodipyrimidine photolyase gene. Their characteristic fried-egg colony morphology was observed on PPLO agar using stereo microscope (Leica, Beijing, China).

The MIC results for the antimicrobials and metals tested in this study were categorised into “low MICs” and “higher MICs” (Antimicrobials such as florphenicol, tetracyclines, and tylosin showed predominantly higher MIC values, with most isolates exhibiting MICs at or above 4 mg/L. Conversely, colloidal silver demonstrated consistently low MICs, with 81.25% of isolates showing MICs at 1.5 mg/L. This categorization highlights the variability in antimicrobial and metal ion efficacy, offering valuable insights into the susceptibility patterns of *Mycoplasma bovis* isolates) based on the distribution of MIC values (Table 2 and Table 3).

The MIC data for the conventional antimicrobials and the metal agents evaluated in this study using (PG45) reference strain were consistent throughout the study and within the range of previous studies, except for tulathromycin, which yielded undetectable MIC values (Table 1).

**Table 1:** Minimum inhibitory concentrations (MICs) and the comparable MIC range tested by others worldwide, as well as the quality control *Mycoplasma bovis* strain PG45, for the tested conventional antimicrobials and metal ions (in mg/L).

Tested Agents	Quality Control Strain *M. bovis* (PG45)	Acceptable Range of MICs Tested by Others *
Phenicol (Florphenicol)	2.00	1.00–32.00
Tetracyclines (chlor- and oxy-tetracycline)	0.25	≤0.12–32.00
Macrolide–tylosin Macrolide–tulathromycin	1.00 0.50	0.06–128.00 ND
Cobalt	3.12	0.78–12.50
Copper	1.56	0.19–12.50
Silver	6.25	0.19–12.50
Zinc	1.56	1.56–12.50
Colloidal silver *	1.56	0.78–1.56

* References [6,34,35,36].

**Table 2:** The minimum inhibitory concentration (MIC) distribution percentages obtained for the 15 *Mycoplasma bovis* isolates against the commonly used conventional antimicrobials.

Tested Antimicrobials	Percentage of *Mycoplasma bovis* Isolates Showing MIC Values at Tested Antimicrobial Concentrations (mg/L)									
	0.25	0.5	1	2	4	8	16	32	64	128
Florphenicol	0	6.25	6.25	18.75	25	43.75	0	0	0	0
Tetracyclines	6.25	6.25	6.25	18.75	0	31.25	25	6.25	0	0
Tulathromycin	0	6.25	12.5	25	25	31.25	0	0	0	0
Tylosin	0	0	6.25	0	37.5	31.25	25	0	0	0

### 3.2. In Vitro Susceptibility Testing

The *M. bovis* susceptibility profile to conventional antimicrobial agents used in this study can be divided as follows:

Florfenicol (a phenicol) and tulathromycin (a macrolide) demonstrated consistently low MICs against *M. bovis*, ranging from 0.5 to 8 mg/L. Both antimicrobials exhibited uniform efficacy. These results are consistent with prior studies, where similar MIC ranges were reported for the PG45 reference strain (Table 1) [6,34,35]. The findings suggest that florfenicol and tulathromycin remain largely effective in inhibiting *M. bovis* at relatively low concentrations, highlighting their continued therapeutic potential.

Higher MICs for tylosin and tetracycline were observed. A tylosin MIC of 16 mg/L was applicable to 25% of the isolates. A small proportion of isolates (6.25%) displayed a high MIC of 32 mg/L to tetracyclines (Table 2). Tetracyclines, particularly oxytetracycline, have been among the most widely used antibiotics, especially for long-term, sequential treatments [37]. This extensive use exerts considerable selective pressure, probably contributing to the development of clinical resistant strains over time. Additional research is needed to confirm these hypotheses while also accounting for environmental influences. A consistent growth was observed in all positive control wells. Nitric acid (solvent) toxicity testing showed no effect on the growth rate of *Mycoplasma* after 24, 48, and 72 hrs of incubation at 37 °C and 5% CO_2_.

The antimicrobial susceptibility of *M. bovis* to conventional antimicrobials showed higher MIC values compared to those reported in a previous Australian study [6]. While the overuse or inappropriate use of antimicrobials in the livestock industry is often suggested as a main factor leading to changes in AMR patterns [38,39], this hypothesis has yet to be proven, particularly when the tested antimicrobials are used in the feedlots where the samples originated.

The metals’ MICs were variable (Table 3). Copper was able to inhibit 6.25% of the isolates at a concentration of 0.1 mg/L, while silver and zinc showed no inhibitory effect at this concentration. Increasing the concentration of copper to 1.5 mg/L caused a 37.5% inhibition of *M. bovis* isolates, and cobalt started showing an effect, inhibiting 12.5% of the isolates. A high percentage (81.25%) of *M. bovis* isolates were inhibited by colloidal silver at a concentration of 1.56 mg/L, while the zinc MIC ranged from 6.2 to 12.5 mg/L. No *Mycoplasma* growth was detected in the microwells at ≥25 mg/L for any of the five tested metals. The MIC_50_ and MIC_90_ were 1.5 mg/L for colloidal silver, or 3 and 6.2 mg/L for copper, and 6.2 and 12.5 mg/L for cobalt, silver, and zinc, respectively.

**Table 3:** The minimum inhibitory concentration (MIC) distribution percentages obtained for the 15 examined *Mycoplasma bovis* isolates against the tested metals.

Tested Metal Agent	Percentage of *Mycoplasma bovis* Isolates Showing MIC Values at the Tested Metal Ion Concentrations (mg/L)									
	0.1	0.3	0.7	1.5	3.1	6.2	12.5	25	50	100
Cobalt	0	0	12.5	0	6.25	56.25	25	0	0	0
Copper	6.25	0	0	37.5	18.75	31.25	6.25	0	0	0
Silver	6.25	0	0	0	6.25	43.75	43.75	0	0	0
Colloidal silver	0	0	18.75	81.25	0	0	0	0	0	0
Zinc	0	0	0	12.5	25	31.25	31.25	0	0	0

In this study, isolates with high MICs (>8 mg/L) to tetracycline and macrolides, but a sensitive response to metals, can be interpreted through several microbiological and genetic mechanisms [39,40]. The observed patterns of MIC values indicated that the regulatory networks conferring AMR do not confer metal resistance. This highlighted the specificity and independence of the AMR mechanisms towards conventional antimicrobials and metals [40,41].

The absence of co-resistance between antimicrobials and metals in this study suggested that selective pressures from conventional antimicrobials had not coincided with or promoted resistance to metals. This may have resulted from different environmental exposures (like the isolates collected from treated cattle) or selective pressures in the bacteria’s natural or clinical settings (for the isolates collected after long exposure to metaphylactic treatment). Unfortunately, these hypotheses could not be confirmed due to a lack of information on the origin and management practices. This differential sensitivity profile is crucial for therapeutic strategies, suggesting that metal-based treatments might be an effective future alternative against *M. bovis* strains resistant to conventional antimicrobials. However, the use of metals in clinical settings must be carefully considered due to their potential toxicity and environmental impacts [39,40].

Low MICs to metals in the current study could be due to an absence or low efficiency of metal efflux systems, which suggests that *M. bovis* might lack effective resistance mechanisms against the cytotoxic effects of these ions [41,42]. It is also possible that the metabolic pathways or structural components of *Mycoplasma* are particularly vulnerable to disruption by metals, leading to cell damage or death even at lower concentrations [43]. The antimicrobial activity of metals against bacteria can be influenced by various factors, including the specific bacterial strain, growth conditions, and the experimental setup. Additionally, the mechanisms of metal interactions are complex and may involve multiple factors such as interference with efflux pumps, modulation of membrane permeability, or direct binding to bacterial components. For instance, copper and zinc have been shown to potentiate the activity of antibiotics by inhibiting efflux pumps and disrupting bacterial membranes [44]. Finally, some of these antibacterial properties may also be harmful to eukaryotic cells, and this should be an area of further research. To the best of our knowledge, this is the first time MICs and time–kill kinetics have been assessed for these metals against *M. bovis* [45]. Overall, the isolates tested in this study showed higher MIC_90_ values for conventional antimicrobials compared to the recent Australian study [6].

### 3.3. Time–Kill Kinetic Assay of Metal Ions

**Figure 1:**
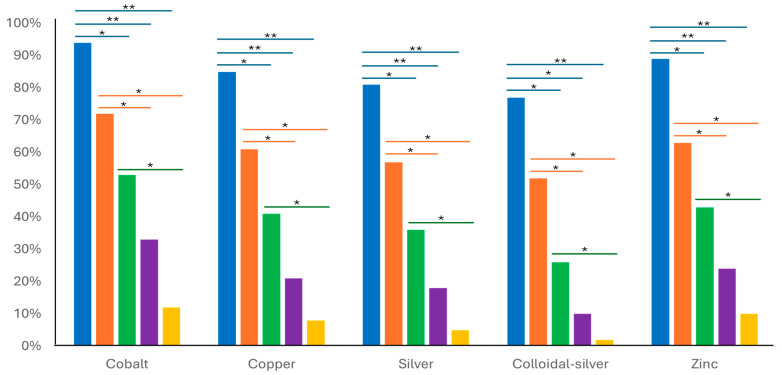
Relative viable count percentages *of Mycoplasma bovis* exposed to different MIC multiples (0.5×, 1×, 2×, 4×, and 8× MIC) for each target metal ion (cobalt, copper, silver, colloidal silver, and zinc) assessed at different points of time–killing: 0 h in blue, 8 h in red, 24 h in green, 48 h in purple, and 72 h in orange-colored bars. A significance of <0.05 down to 0.001 is marked by a single asterisk (*), and significance <0.001 is marked by a double asterisk (**). Blue Bars (0 h): The blue bars indicate the baseline viable count of *M. bovis* at (0 h), before exposure to the tested metal ions. These bars provide a reference point for comparing bacterial survival over time and across different concentrations of metals. Yellow Bars (72 h): The yellow bars represent the viable count after 72 h of exposure to the tested metal ions at the specified MIC multiples. These bars illustrate the cumulative bactericidal effect over the entire incubation period.

The time–kill measured across different points (0 h, 8 h, 24 h, 48 h, and 72 h) showed a reduction in *M. bovis* when treated with different ascending concentrations (0.5× MIC, 1× MIC, 2× MIC, 4× MIC, and 8× MIC) of metals (Figure 1). For example, cobalt and zinc demonstrated a pronounced bactericidal effect on *M. bovis* at 2× MIC, with the viable count being reduced by 50%, increasing to 73% at 4× MIC, and achieving an 88% reduction at 8× MIC within 24 h or longer. Silver and its colloidal alloy showed good effectiveness at 2× MIC, achieving a reduction of 63%, which increased to 73% and almost 90% at 4× MIC after 48 hrs. The bactericidal endpoint, where no viable *M. bovis* cells could be detected, was reached after 72 h of incubation at 8× MIC (12 mg/L) for colloidal silver. In contrast, cobalt and zinc did not consistently reach these bactericidal endpoints within the same time frame (Figure 1).

Our results showed that metals of colloidal silver and copper showed the most promising antimicrobial properties (Table 2). Similarly, the time–kill kinetics of colloidal silver and copper were indicative of the highest efficacy against *M. bovis*. The time–kill kinetics revealed a combination of concentration-dependent and time-dependant activity for all metals. Overall, the current study suggests that a metal may have some potential in the management of *M. bovis*, but further work is required before a definitive conclusion can be drawn. Interestingly, strains resistant to conventional antimicrobials (e.g., tetracyclines) had no resistance to metals, particularly colloidal silver.

The time–kill curves demonstrated that the bactericidal effects of metals are concentration- and time-dependent. Higher concentrations of metal ions generally resulted in faster and more complete inhibition of *M. bovis* growth. Colloidal silver exhibited the highest bactericidal effect, achieving complete inhibition at lower concentrations (4× MIC) and at the earliest time points (24 h) compared to other metals. Cobalt and copper also showed strong inhibition, particularly at higher concentrations. These observations suggest that while silver and copper are highly effective in reducing the viability of *M. bovis* over a shorter duration, the effectiveness of cobalt and zinc, particularly at higher concentrations, may be influenced by factors not captured in this dataset [46].

The *p*-value from the ANOVA was 0.05, indicating no difference in the inhibition effects of antimicrobials or metals. The results of the current study supported the hypothesis that the differential resistance profiles of conventional antimicrobials and metals highlight distinct microbiological adaptations within *Mycoplasma*. Tetracycline and macrolides, which inhibit protein synthesis by targeting ribosomal subunits, often encounter resistance through mechanisms like efflux pumps, ribosomal protection proteins, and less commonly, enzymatic inactivation [47].

This study has some limitations. One important limitation is the relatively small sample size. However, despite the study’s small sample size, the reliability of the findings was supported by the consistency in susceptibility of the reference strain, and the close alignment with the existing literature [48]. Another limitation is the lack of interpretive criteria for metals and some antimicrobials for *M. bovis*. Overall, metals showed similar or higher MIC_90_ compared to the tested conventional antimicrobials. Therefore, these concentrations are likely achievable in the biological medium of the host. However, as we have not carried out tests for the transition from viable to viable-but-not-culturable status, like most other studies, we cannot confirm that the *M. bovis* isolates did not undergo such a change.

The exploration of in vitro metal antimicrobial activity against veterinary *Mycoplasma* is an essential step in understanding the potential usefulness of these metals and/or their alloys in the treatment of infections caused by these pathogens. Despite the advantages of metals, metal-based compounds face challenges, including limited research on their pharmacological behavior and toxicity thresholds. High dosages of metal ions may exacerbate in vivo toxicity, as mammalian cells share similar targets with bacteria. Moreover, metals not naturally occurring as trace elements in the human body may pose toxicity risks. Finally, the potential environmental impact of heavy metal ions was not considered in this study. Addressing these concerns requires further in vivo and clinical studies to determine safe and effective routes of administration while ensuring their therapeutic potential [26,49,50].

Further in vitro and in vivo clinical studies are necessary to evaluate metal ions’ safety, optimal dosage, and therapeutic potential in animals and humans. To ensure effective and safe use, this additional research should determine their pharmacokinetics, toxicity thresholds, and practical applications.

## 4. Conclusions

Our results demonstrated that colloidal silver and copper exhibited inhibitory effects on tested *M. bovis* strains, with colloidal silver showing notable potential against tetracycline-resistant strains. While some metal ions displayed higher inhibition rates, the variability in responses limited their significance, except in time–kill kinetics assays. Despite this, the observed biological significance highlights the potential of these agents as effective alternatives in combating antimicrobial resistance. Integrating metal-based compounds into existing treatment strategies could provide promising options for veterinary medicine. However, further comprehensive research is needed to fully explore their therapeutic potential and address the broader challenges of antimicrobial resistance.

## Data Availability

A total of 15 isolates collected either from lung samples (swab, fresh tissue, or both) were obtained during 2021–2023 from post-mortem BRD-affected cattle in different states in Australia: Queensland (QLD), New South Wales (NSW), and South Australia (SA). Further details are provided in our previous publication 5 (Hasoon et al., 2023 [6]). These isolates were obtained from animals that had been previously treated with antimicrobials but failed to respond.
